# Pharmacological Strategies for Pain Relief in Patients with Terminal Delirium: A Secondary Data Analysis

**DOI:** 10.1177/26892820251390525

**Published:** 2025-10-24

**Authors:** Takaaki Hasegawa, Masanori Mori, Takashi Yamaguchi, Kengo Imai, Yoshinobu Matsuda, Isseki Maeda, Yutaka Hatano, Hiroto Ishiki, Hiroyuki Otani, the East Asian collaborative cross-cultural study to elucidate the drying process (EASED) investigators

**Affiliations:** ^1^ Center for Psycho-oncology and Palliative Care, Nagoya City University Hospital, Nagoya, Japan.; ^2^ Palliative and Supportive Care Division, Seirei Mikatahara General Hospital, Hamamatsu, Japan.; ^3^ Department of Palliative Care, Konan Medical Center, Kobe, Japan.; ^4^ Seirei Hospice, Seirei Mikatahara General Hospital, Hamamatsu, Japan.; ^5^ Department of Psychosomatic Internal Medicine, NHO Kinki Chuo Chest Medical Center, Sakai, Japan.; ^6^ Department of Palliative Care, Senri-Chuo Hospital, Toyonaka, Japan.; ^7^ Department of Palliative Care, Daini Kyoritsu Hospital, Kawanishi, Japan.; ^8^ Department of Palliative Medicine, National Cancer Center Hospital, Tokyo, Japan.; ^9^ Department of Palliative Care Team, Palliative and Supportive Care, National Hospital Organization Kyushu Cancer Center, Fukuoka, Japan.; ^10^ Department of Palliative Care Team, Palliative and Supportive Care, St. Mary’s Hospital, Kurume, Japan.

**Keywords:** neoplasms, pain, delirium, palliative care, opioid, antipsychotic agents

## Abstract

**Background:**

Terminally ill cancer patients often experience pain and delirium. However, opioids administered for pain management may exacerbate patients’ delirium.

**Objectives:**

To explore the real-world symptom trajectory associated with pharmacological interventions, including opioids and antipsychotics, in patients with cancer pain and terminal delirium.

**Design:**

A secondary analysis of a multicenter prospective observational study.

**Setting/Subjects:**

Adult patients admitted to inpatient hospice or palliative care units in Japan. Participants were eligible if they had cancer pain (Integrated Palliative care Outcome Scale: IPOS ≥2) and delirium at the time that their Palliative Performance Scale had declined to ≤20 (day 1, immediately before death).

**Measurements:**

Pharmacological strategies, pain levels (using the IPOS), and delirium symptoms (using the Memorial Delirium Assessment Scale, item-9).

**Results:**

Among a total of 1896 patients, 1396 were assessed for eligibility on day 1, and 137 met the inclusion criteria for analysis. A total of 86 (63%) patients had agitated delirium (hyperactive or mixed) with a median survival time of three days. Regarding pharmacological strategies, 32 (23%) received opioid initiation/dose escalation and 94 (69%) received regular administration of antipsychotics. These figures also included 25 (18%) patients who received both opioid initiation/dose escalation and antipsychotics. Approximately 55% of all patients had persistent cancer pain (IPOS for pain ≥2) on day 2. Among those with agitated delirium, 79% continued to exhibit agitation symptoms on day 2.

**Conclusion:**

Despite specialized palliative care, the combined distress of cancer pain and delirium in the last days of life remains complex and refractory.

## Key Message

This multicenter prospective observational study examined pain and delirium in cancer patients receiving specialized palliative care. Findings suggest that symptoms of pain and delirium result in complex refractory suffering, with pharmacological interventions of opioids and antipsychotics offering limited efficacy, highlighting the need for improved symptom management strategies in end-of-life care.

## Introduction

Cancer pain is a prevalent and distressing symptom in patients with advanced cancer, affecting 35%–96% of individuals.^1^ Opioids are the cornerstone of cancer pain management. When initial opioid therapy provides inadequate relief, dose titration is employed to ensure adequate pain control. However, opioid-induced delirium is a significant concern in terminally ill patients experiencing cancer pain.^2^

Delirium is also a common symptom, with 42%–88% of terminally ill cancer patients experiencing delirium.^3^ The use of antipsychotics remains an option in the management of terminal delirium with agitation.^4,5^ As delirium can amplify pain perception, there is an ongoing debate regarding whether antipsychotics should be prioritized in cases of coexisting delirium and pain or whether analgesia should be administered first, despite the potential risk of exacerbating delirium.

This preliminary hypothesis-generating study focuses on cancer pain complicated by delirium in terminally ill patients. We examined pharmacological strategies—the use of opioids and antipsychotics—for managing pain in patients with delirium and assessed the symptom trajectory of both pain and delirium during treatment. We focused on delirium, especially terminal delirium that occurs in the dying phase with the implication that reversal will not be pursued.^6^

## Methods

This study is based on a secondary analysis of a multicenter prospective study investigating some aspects of the dying process and end-of-life care.^7^ The institutional review board at each participating site approved the study protocol. These participating sites have been previously listed.^8^ Typically, palliative care units (inpatient hospice) in Japan form part of general hospitals and provide end-of-life care for patients with advanced cancer. They are usually built separately from the general ward or on different floors. For certification, they are required to fulfill regulations in terms of space, presence of private rooms, availability of palliative care physicians, and equipment for families. This study was conducted in accordance with the principles of the Declaration of Helsinki. As per Japanese ethical guidelines,^9^ we used an opt-out method rather than acquiring written or oral informed consent. All patients in this noninvasive observational study could receive information on the study through instructions posted on the ward or institutional website and consent was presumed if the patients or their caregivers did not actively refuse to give consent for the reuse of data.

### Participants

Patients were enrolled between January and December 2017. Adult patients (≥18 years) with locally advanced or metastatic cancer were recruited upon admission to the inpatient hospices or palliative care units (in 23 facilities). Patients were included in this secondary analysis if they: (a) had a pain score ≥2 on the Integrated Palliative Outcome Scale (IPOS), and (b) had delirium (using the 5th edition of the Diagnostic and Statistical Manual of Mental Disorders) at the time of showing Palliative Performance Scale (PPS) ≤20 (i.e., totally bed bound, requiring full assistance, and minimal oral intake, limited to sips or mouth care only).

### Measurement

#### Pharmacological strategy

To assess the use of opioid treatment, data on opioid administration and the oral morphine equivalent daily dose were collected on day 1 (when PPS ≤20), and day 2. Additionally, the use of antipsychotics on day 1 was also recorded. On day 2, initiation of new opioids for opioid-naïve patients or dose escalation from day 1 was defined as *opioid initiation/dose escalation*. The pharmacological strategy, including opioid initiation/dose escalation and regular administration of antipsychotics, was categorized into four types: *no opioid initiation/dose escalation without antipsychotics*, *opioid initiation/dose escalation without antipsychotics*, *no opioid initiation/dose escalation with antipsychotics*, and *opioid initiation/dose escalation with antipsychotics*.

#### Pain

Pain was evaluated using the staff proxy version of the IPOS for the worst pain experienced over 24 hours. The IPOS is a validated scale widely used for assessing pain in patients receiving palliative care. IPOS items are rated on a 5-point Likert scale (0 = not at all, 1 = slightly, 2 = moderately, 3 = severely, and 4 = overwhelmingly).^10^ Cases in which the IPOS for pain score could not be assessed owing to a loss of consciousness (e.g., initiation of sedation or the natural dying process) were recorded as “cannot assess.”

#### Delirium

At enrollment (when PPS ≤20), delirium subtypes were categorized as hyperactive, mixed, and hypoactive. The severity of delirium symptoms was assessed using item 9 of the Memorial Delirium Assessment Scale (MDAS; rated 0 = none, 1 = mild, 2 = moderate, 3 = severe),^11^ measuring the worst agitation level over 24 hours; item 4 of the Communication Capacity Scale (CCS, rated 0 to 3, with lower scores indicating better communication capacity),^12^ assessing the best communication ability over 24 hours; and the modified Richmond Agitation-Sedation Scale (RASS, scored from −5 to +4),^13^ evaluated on both days 1 and 2.

### Statistical analysis

This analysis focused on pharmacological strategies for managing pain in patients with delirium. The analysis also assessed changes in both pain and delirium symptoms from day 1 to day 2 using a paired *t*-test. We categorized patients’ pain into three groups: “no/mild pain” (IPOS pain score ≤1 on day 2), “persisting pain” (IPOS pain score ≥2 on day 2), and “unable to assess pain” (IPOS pain score not available on day 2). Statistical analyses were conducted using SPSS v.28.0 (IBM Corp., Armonk, NY, USA).

## Results

Among the total of 1896 patients in the primary study, data obtained on day 1 were available for 1396 and only these patients were assessed for eligibility. Of these, 1227 patients were excluded for the following (overlapping) reasons: no delirium (n = 653), no pain (n = 782), and unable to assess pain (n = 328). Of the remaining 169 patients, 32 were further excluded due to missing data on the pharmacological treatment strategy. Finally, 137 patients were included in the secondary analysis.

The patients’ mean age was 68 years (standard deviation: 12.3), with 42% being female. The most common primary cancer site was the gastrointestinal tract (31%); 39% of the patients had bone metastasis, 29% regularly used corticosteroids, and 93% were opioid-tolerant. The median survival time was 3 days (interquartile range: 2–7).

Regarding pharmacological strategies, 69 (50%) patients received *no opioid initiation/dose escalation with antipsychotics*, 36 (26%) patients received *no opioid initiation/dose escalation without antipsychotics*, 25 (18%) patients received *opioid initiation/dose escalation with antipsychotics*, and 7 (5%) patients received *opioid initiation/dose escalation without antipsychotics*.

### Pain intensity by pharmacological strategy

**FIG. 1.**
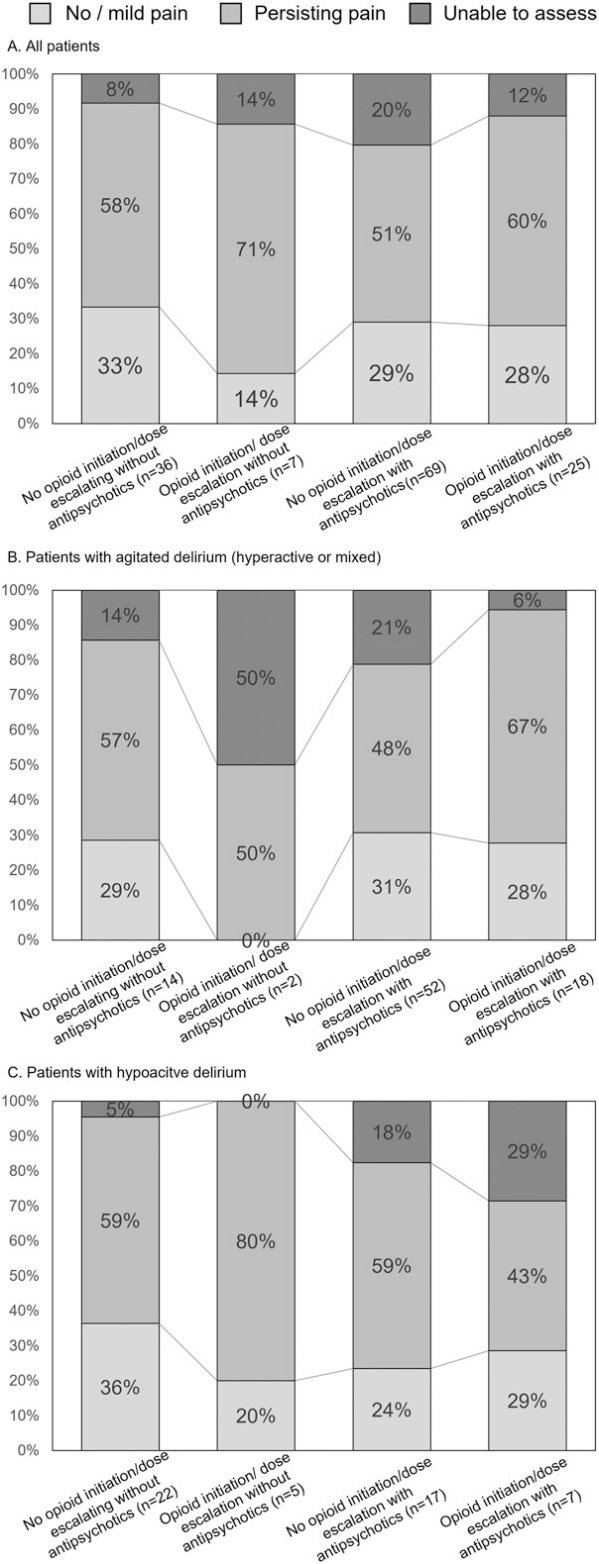
The proportion of patients on the basis of longitudinal changes in pain intensity (IPOS) on day 2. IPOS: Integrated Palliative Outcome Scale; no/mild pain: IPOS for pain <2; persisting pain: IPOS for pain ≥2; unable to assess pain: no available responses using the IPOS for pain on day 2 were categorized as “cannot assess.”

In terms of pain intensity, the mean IPOS score for pain decreased from day 1 to day 2 (0.50, 95% confidence interval [CI]: 0.35–0.65). The proportion of patients with “no/mild pain” and “persisting pain” (IPOS for pain ≥2) on day 2 was 29% (40/137) and 55% (76/137), respectively. Figure 1 shows the proportion of patients on the basis of longitudinal changes in pain intensity (IPOS) on day 2 by pharmacological strategy. The proportion of such patients with “no/mild pain” by pharmacological strategy was as follows: 33% (12/36, 95% CI: 20–50) in the *no opioid initiation/dose escalation without antipsychotics* group, 14% (1/7, 95% CI: 1–53) in the *opioid initiation/dose escalation without antipsychotics* group, 29% (20/69, 95% CI: 20–41) in the *no opioid initiation/dose escalation with antipsychotics* group, and 28% (7/25, 95% CI: 14–48) in the *opioid initiation/dose escalation with antipsychotics* group.

### Delirium symptoms

On day 1, agitated delirium (hyperactive or mixed) was observed in 86 patients (63%). In terms of the severity of agitation, the mean MDAS item 9 score decreased from day 1 to day 2 (0.22; 95% CI: 0.11–0.33). In the agitated delirium group, the proportion of patients with remaining agitation symptoms (MDAS item 9 ≥ 1) was 79% (68/86). Communication capacity, measured using CCS item 4, significantly deteriorated from day 1 to day 2 (−0.24, 95% CI: −0.36 to −0.13). The RASS scores indicated deeper sedation levels from day 1 to day 2 (0.75; 95% CI: 0.51–0.99).

### Palliative sedation

The proportions of patients who received intermittent and continuous palliative sedation were 16% (22/137) and 10% (14/137), respectively.

## Discussion

In this secondary analysis of a nationwide study, we examined real-world practices for managing pain and delirium symptoms among patients receiving inpatient-specialized palliative care services. We also analyzed the symptom trajectory of cancer pain complicated by delirium under different pharmacological strategies.

For patients experiencing moderate-to-severe cancer pain complicated by delirium, immediately before their death, the predominant opioid strategy in inpatient-specialized palliative care services was no opioid initiation or dose escalation (77%: 105/137). On day 1 (when PPS ≤20), 93% of the patients had already been receiving opioids. This suggests that palliative care physicians may have refrained from opioid dose escalation due to concerns about worsening delirium or because the pain was deemed refractory to opioid treatment.

More than half of the patients continued to experience persistent pain (IPOS pain ≥2) on day 2. This aligns with previous research suggesting that pain in the final week of life often remains under-treated, even when the patient receives specialized palliative care.^14,15^ Similar challenges exist in the management of terminal dyspnea combined with delirium in patients with advanced cancer.^16^ However, specific intervention strategies for managing complex end-of-life symptoms remain to be designed. Given the complexity of these overlapping symptoms, establishing treatment algorithms for terminal delirium and refractory pain is necessary,^11,12^ as well as integration of a comprehensive symptom management approach involving opioids, antipsychotics, and palliative sedation.

This study has several limitations. First, cancer pain intensity was assessed based on physician-reported data rather than patient-reported outcomes (e.g., numerical rating scale). However, validated patient-reported pain assessment instruments are currently lacking for patients with delirium.^17^ While tools such as the Pain Assessment in Advanced Dementia scale are available, they have not been widely utilized. The staff proxy version of the IPOS for pain remains a reasonable alternative for assessing patients with terminal delirium. Second, owing to the secondary nature of this analysis, we were unable to assess the direct efficacy of pharmacological treatment, including opioids and antipsychotics, while controlling for co-interventions such as sedatives and nonpharmacological treatments. Third, as this study was conducted in specialized Japanese palliative care units, the conclusions may have limited generalizability owing to differences in end-of-life care practices. Fourth, many patients were excluded, including those with delirium and those recorded as “unable to assess pain.” The category “cannot assess” is included among the definitions in the IPOS, which may have resulted in the exclusion of potentially eligible cases even though the staff proxy version of the IPOS was used. Given these several limitations, the results of this study need to be confirmed in a prospective observational study using a pain assessment tool such as the Non-communicative Patient’s Pain Assessment Instrument.

In conclusion, the primary approach to managing cancer pain complicated by terminal delirium is to avoid initiating or escalating opioid doses. Additionally, the combination of cancer pain and delirium in the final days of life involves complex, refractory suffering, with pharmacological interventions showing limited efficacy. Further research is needed to develop a comprehensive treatment strategy that integrates symptom-specific relief algorithms and sedative interventions to optimize care for patients with cancer pain complicated by terminal delirium.

## Data Availability

The data supporting the results of this study are available from the corresponding author upon reasonable request.

## Abbreviations Used

IPS: Integrated Palliative care Outcome Scale
PPS: Palliative Performance Scale
MDAS: Memorial Delirium Assessment Scale
CCS: Communication Capacity Scale
RASS: Richmond Agitation-Sedation Scale
